# Evaluation of the analytical and clinical characteristics of the siemens immulite®2000 tsi method for determining thyrotropin receptor antibodies

**DOI:** 10.5937/jomb0-55873

**Published:** 2025-06-13

**Authors:** Neda Milinković, Marija Sarić, Ana Ružanović, Miloš Žarković, Jasmina Ćirić, Iva Perović Blagojević, Ana-Marija Mastilović, Svetlana Ignjatović, Biljana Nedeljković Beleslin

**Affiliations:** 1 University of Belgrade, Faculty of Pharmacy, Department of Medical Biochemistry, Belgrade; 2 University Clinical Center of Serbia, Clinic of Endocrinology, Belgrade, Serbia; 3 University of Belgrade, Medical Faculty, Belgrade; 4 Clinical Hospital Center "Dr Dragiša Mišović-Dedinje", Service for laboratory diagnostics, Belgrade

**Keywords:** thyroid stimulating hormone receptor antibodies, analytical and clinical sensitivity and specificity, antitela na receptore stimulišućeg hormona štitne žlezde, analitička i klinička osetljivost i specifičnost

## Abstract

**Background:**

Despite commercially improved, standardised routine methods used in medical laboratories, precision laboratory medicine lacks harmonisation of results to make the laboratory result useful for its intended purpose. Furthermore, to obtain reliable laboratory results and precise diagnoses, it is important and recommended that each laboratory confirms the analytical and clinical characteristics of the method used. This study aimed to evaluate the analytical and clinical performance of the IMMULITE®2000 TSI bridge immunoassay to determine autoreactive thyroid stimulating hormone receptor antibodies (SH-R-Ab).

**Methods:**

A total of 86 patients with clinically present Graves' orbitopathy and 23 healthy volunteers as a control group were included in the study. The total TSH-R-Ab concentration was determined using an ECLIA (Elecsys Anti-TSHR Immunoassay Roche Diagnostics, GmbH, Mannheim, Germany) on the Cobas e411 analyser (Roche, Diagnostics, GmbH). The TSH-R-Ab concentration was measured using a CLIA method (IMMULITE TSI 2000, Siemens Healthcare Diagnostics, UK). The inaccuracy of the method was investigated using two levels of commercial control samples (low and high analyte concentration).

**Results:**

The results obtained meet the general minimum requirements for the analytical performance of laboratory methods (CV<5%). The overall laboratory inaccuracy was acceptable according to FDA guidelines (CV<20%). The results showed a statistically significant correlation between the analysed methods (r=0.9041, p < 0.0001) but with a relative bias of 24.5%. The best ratio of sensitivity and specificity determined by the ROC analysis (93.3% and 100%, respectively) was obtained for a cut-off value of 0.1215 IU/L, which is significantly lower compared to the cut-off value specified by the manufacturer (0.55 IU/L).

**Conclusions:**

The IMMULITE 2000 TSI bridge immunoassay for TSH-R-Ab quantification confirmed adequate precision, which is essential for routine use. However, further studies are required to evaluate its analytical specificity.

## Abstract (sr)

**Uvod:**

Uprkos komercijalno poboljšanim, standardizovanim rutinskim metodama koje se koriste u medicinskim laboratorijama, preciznoj laboratorijskoj medicini nedostaje harmonizacija rezultata kako bi laboratorijski rezultat bio koristan za njegovu namenu. Dodatno, za dobijanje pouzdanih laboratorijskih rezultata i preciznijih dijagnoza važno je i preporučuje se da svaka laboratorija potvrdi analitičke i kliničke karakteristike primenjene metode. Cilj ove studije bio je da se procene analitičke i kliničke performanse IMMULITE®2000 TSI imunohemijskog testa za odre|ivanje autoreaktivnih antitela na receptore hormona koji stimulišu štitnu žlezdu (TSH-R-Ab).

**Metode:**

U studiju je uključeno ukupno 86 bolesnika sa klinički prisutnom Gravesovom orbitopatijom i 23 zdrava dobro voljca kao kontrolna grupa. Ukupna koncentracija TSH-R-Ab određena je pomoću ECLIA (Elecsys Anti-TSHR Immunoassay Roche Diagnostics, GmbH, Mannheim, Germany) na analizatoru Cobas e411 (Roche, Diagnostics, GmbH). Koncentracija TSH-R-Ab merena je metodom CLIA (IMMULITE TSI 2000, Siemens Healthcare Diag - nostics, UK). Netačnost metode ispitana je pomoću dva nivoa komercijalnih kontrolnih uzoraka (niska i visoka koncentracija analita).

**Rezultati:**

Dobijeni rezultati ispunjavaju opšte minimalne zahteve za analitičke performanse laboratorijskih metoda. (CV<5%). Ukupna laboratorijska netačnost bila je prihvatljiva prema smernicama FDA (CV<20%). Rezultati su pokazali statistički značajnu korelaciju izme|u analiziranih metoda (r=0,9041, p<0,0001), ali sa relativnim odstupanjem od 24,5%. Najbolji odnos osetljivosti i specifičnosti utvrđen ROC analizom (93,3% odnosno 100%) dobijen je za graničnu vrednost od 0,1215 IU/L, što je značajno niže u poređenju sa graničnom vrednošću koju je odredio proizvođač (0,55 IU/L).

**Zaključak:**

IMMULITE 2000 TSI imunohemijski test za kvantifikaciju TSH-R-Ab potvrdio je adekvatan nivo preciznosti koji je neophodan za rutinsku upotrebu. Međutim, potrebna su dalja istraživanja kako bi se procenila njegova analitička specifičnost.

## Introduction

Autoreactive thyroid-stimulating hormone receptor antibodies ( SH-R-Ab) are key laboratory tools in the diagnosis and follow-up of Graves’ disease (GD) and its most common extrathyroidal manifestation, Graves’ orbitopathy (GO) [1] [2] [3]. Both GD and GO are chronic diseases characterised by periods of remission and relapses, with severe forms rarely achieving a complete recovery. GO is a multifactorial autoimmune disease with insufficiently described aetiology and pathogenesis. In severe cases, it can lead to dysthyroid optic neuropathy, a medical emergency with the risk of permanent blindness that requires immediate intervention [4] [5] [6]. Early detection by serological testing is crucial for timely diagnosis, therapy optimisation and reliable monitoring of both diseases [3].

TSH-R-Ab express the heterogeneous biological activity and are classified as stimulating (TSAb), blocking (TBAb), and neutralising antibodies [7]. This property has been exploited to develop analytical methods and recognised as fundamental for further research into thyroid and orbital autoimmunity [7] [8] [9]. The functional fraction of these antibodies does not destroy the target cells but interferes with their metabolism and consequently impairs their function [10]. TSAb stimulate cAMP-mediated signalling and cause hyperthyroidism and orbitopathy, while TBAb block the cAMP signalling pathway causing hypothyroidism [10] [11] [12]. TSH-R-Ab that bind to the TSH-R without affecting the natural ligand binding and cAMP signalling are referred to as neutral, and their exact role is still unknown. There is evidence that this class of antibodies exerts its effect by inducing alternative signalling pathways that eventually lead to apoptosis [13].

The methodology for determining TSH-R-Ab has undergone considerable technical and analytical improvements over time [14] [15] [16]. Despite developing and applying more sophisticated bioassay technologies that can successfully quantify the functionality of TSH-R-Ab, routine laboratory testing still mainly uses immunochemical methods to measure TSH-R-Ab [16]. Competitive binding immunoassays are the most commonly used tests in routine laboratory diagnostics. They measure the total concentration of TSH-R-Ab, commonly referred to as TSH receptorbinding inhibitory immunoglobulins (TBII), which is the net sum of different TSH-R-Ab types but does not reflect their biological activity [7] [17] [18]. Today, these assays mostly employ chemiluminescent labelled monoclonal antibodies that compete with TSH-R-Ab from patients’ sera for the same binding sites on the TSH-R [14]. Recently, another immunoassay has been introduced into practice and is intended for application on fully automated commercial platforms. It utilises bridge immunoassay technology and was reportedly developed to measure the concentration of TSAb [15] precisely.

The application of standard reference materials and the use of monoclonal TSH-R-Ab as commercially available calibrators has significantly improved the accuracy of determination [19] [20]. The turnaround time could be considerably shortened, as the analysis takes up to 2 hours. Significant diagnostic sensitivity and specificity ( 90%) were achieved for the differential diagnosis of GD compared to other causes of hyperthyroidism [21]. The advantages and disadvantages of competitive binding assays compared to bioassays are still under investigation [22]. Still, the possibility of routine use of immunoassays requires optimisation of the methodology and further improvements in analytical and clinical properties.

This study aimed to evaluate the analytical and clinical performance of the IMMULITE®2000 TSI bridge immunoassay to determine TSH-R-Ab. For this purpose, we examined the method’s repeatability and reproducibility, as well as the accuracy and systematic error regarding the traditional competitive binding assay. In addition, the diagnostic characteristics of the IMMULITE®2000 TSI were examined.

## Materials and methods

### Study participants

A total of 86 patients who were regularly treated at the Clinic for Endocrinology, Diabetes, and Metabolic Diseases at the University Clinical Center of Serbia were included in the study. The main inclusion criterion was a clinically present GO, while the main exclusion criterion was the administration of corticosteroids or other immunosuppressive drugs for at least six months before enrollment. All patients underwent endocrinologic and ophthalmologic examination, thyroid ultrasonography, orbital ultrasonography, computed tomography, routine laboratory analysis of thyroid function, and thyroid autoimmunity (thyroid function tests). Patients were categorised according to their thyroid status into patients with GD and GO, patients with Hashimoto’s thyroiditis (HT) and GO, and patients with a euthyroid form of GO.

All diagnostic and therapeutic procedures followed the guidelines of the National Guide of Good Clinical Practice for Thyroid Disorders [23], as well as the guidelines of the European Thyroid Association (ETA) [24] and the European Group of Graves’ Orbitopathy (EUGOGO) organisations [25].

The activity of GO was assessed based on the clinical activity score (CAS), whereby GO was classified as active or inactive (seven points Clinical Activity Score (CAS), cut-off 3/7) [26]. The severity of GO was classified as mild, moderate to severe GO, and sight-threatening GO based on EUGOGO recommendations [25].

In order to evaluate the clinical characteristics of the new IMMULITE TSI 2000 bridge assay, we additionally included 23 healthy volunteers in the study as a healthy control group. The criterion for including healthy subjects in the study was the absence of any thyroid and inflammatory diseases, confirmed by routine laboratory analyses and standard questionnaires. Exclusion criteria were a positive history of thyroid disease, any other autoimmune and endocrine diseases and any type of immunosuppressive therapy.

The patients’ data were obtained from the hospital database, and their samples were collected using a standardised protocol. For this study, all participants signed an informed consent form before the start of the study. The study was conducted in accordance with clinical and laboratory practice guidelines, the Declaration of Helsinki and applicable institutional and national regulations. The Ethics Committee of the Faculty of Pharmacy, University of Belgrade, previously approved the conduct of this study (June 17, 2019/944/3).

### Blood draw

The blood draw was performed in the morning, after a 12-hour fasting period. A venous blood sample was collected by a standardised venipuncture procedure. To obtain serum, venous blood samples were collected in a vacutainer without anticoagulant, with serum separation gel (Becton, Dickinson, BD Vacutainer® SST™ Tubes). All samples were triaged by a standard centrifugation procedure. After the separation of serum, samples were appropriately aliquoted and stored at -80°C until analysis.

### Methods

### Routine competitive binding immunoassay

Total TSH-R-Ab concentration was determined by the use of a commercial competitive binding ECLIA immunoassay (Elecsys Anti-TSHR Immunoassay Roche Diagnostics, GmbH, Mannheim,Germany) on the Cobas e411 analyser (Roche, Diagnostics, GmbH) according to the manufacturer’s instructions.

### Bridge immunoassay

In addition, the TSH-R-Ab concentration was measured with a commercial immunochemical CLIA method that uses the principle of determination in the bridge assay format (IMMULITE TSI 2000, Siemens Healthcare Diagnostics, UK). According to the manufacturer’s instructions, the analysis was performed in the Laboratory Diagnostic Service of the Clinical Hospital Center »Dr. Dragiša Mišović-Dedinje« in Belgrade, using a Siemens IMMULITE 2000 analyser.

### Analytical characteristics evaluation

As part of verifying the analytical performance of the commercial IMMULITE® 2000 TSI bridge assay, the following characteristics were analysed: within-run and between-run imprecision, repeatability, reproducibility, within laboratory imprecision and accuracy of the method. The imprecision of the method was examined using two levels of commercial control samples (low and high analyte concentration). The indicated median values and interquartile range (IQR) for Control Level 1 and Control Level 2 are 0.88 IU/L (0.78–0.98) and 18.4 IU/L (16.6–20.2), respectively. The analytical evaluation procedure for precision and accuracy was conducted according to the actual guidelines of the Clinical Laboratory Standards Institute [27] [28]. The calculated coefficients of variation (CV) were compared with those the manufacturer declared. If the value of the calculated CV was lower than the one defined by the manufacturer, we may conclude that the method meets the predefined requirements for precision. If the manufacturer has not defined the minimum criteria for imprecision, the CV values were compared with the values defined by the Food and Drug Administration (FDA) for that type of analytical method [29]. As the general minimum of the analytical performance of laboratory analytical methods, the CV value of 5% was considered.

### Within run imprecision

Within run imprecision was examined by consecutively analysing two levels of control samples 20 times in one run and calculating the following statistical parameters: mean value (x̄), standard deviation (Sd) and CV.

### Between run imprecision

Between run imprecision was examined by repeated determinations of 10 times in one run and 10 times in the second for two levels of control samples, in the low and high range of concentrations. Subsequently, the following statistical parameters were calculated: mean value (x̄), standard deviation (Sd) and CV.

### Repeatability of the measurement

The measurement repeatability was calculated according to the CLSI EP15-A2 guidelines.

### Day-to-day imprecision

Day-to-day imprecision was examined by consecutively measuring two levels of control samples in triplicate for 5 days. After that, the following statistical parameters were calculated: mean value (x̄), standard deviation (Sd) and CV.

### Within laboratory imprecision

Within laboratory measurement imprecision within the laboratory and overall laboratory imprecision for the IMMULITE®2000 TSI method was calculated according to CLSI EP15-A2 guidelines by a combined analysis of day-to-day imprecision and repeatability of method measurements. The obtained CV value was compared with the one defined by the manufacturer of the test for each control level.

Repeatability and reproducibility for the IMMULITE®2000 TSI method were tested by loading one run in one series for each control sample and loading both control samples five consecutive days in triplicate, respectively.

### Clinical characteristics evaluation

As part of the evaluation of the clinical performance of the method, the following clinical characteristics were determined: diagnostic sensitivity, diagnostic specificity, positive predictive value (PPV), negative predictive value (NPV), and ROC analysis (Receiver Operating Characteristic).

### Statistical analysis

Statistical analysis of all data obtained in this work was performed using Excel (version 2010; Microsoft, USA) and statistical programs SPSS (IBM® SPSS® Statistics version 20, IBM, USA) and MedCalc (version 12; MedCalc Software, Belgium). The normality of data distribution was tested using Kolmogorov-Smirnov and Shapiro-Wilk statistical tests, depending on the amount of data in the group and the statistical program used. Continuous variables are presented as mean value and standard deviation (x̄±Sd), and medians and interquartile range. Normally distributed continuous variables were compared using the Student-t test for repeated measurements and non-parametric data using the Wilcoxon signed-rank test. Spearman’s non-parametric correlation analysis was used to examine the level of correlation. Passing Bablock analysis was used for method comparison. The process of analytical evaluation and verification of the precision and accuracy of the analysed methods was carried out following the updated CLSI documents [27] [28]. Statistical significance was assumed at a value of p<0.05.

Diagnostic accuracy of the investigated analytical methods was carried out using contingency tables (calculations of the sensitivity, specificity, positive and negative predictive value) and ROC analysis. Area under the curve (AUC) values in the range of 0.90 to 1 defined excellent diagnostic accuracy, values of 0.80 to 0.90 defined good diagnostic accuracy, values of 0.70 to 0.80 moderate diagnostic accuracy, and values from 0.6 to 0.70 indicated poor diagnostic accuracy. For AUC values of 0.5 to 0.6, it was considered that the criteria of minimum acceptable diagnostic accuracy were not met.

## Results

The values of CV for within-run and betweenrun imprecision are presented in Table 1. Theobtained values meet the general minimum requirements for the analytical performance of laboratory methods (CV<5%). Both within and between-run variations were acceptable according to FDA guidelines (CV<20%) [29].

**Table 1:** Within-run and Between-run imprecision. x̄ - mean value; SD – standard deviation; CV – coefficient of variance.

Control level	Within-run imprecision		Between-run imprecision	
	x̄ ± SD (IU/L)	CV (%)	x̄ ± SD (IU/L)	CV (%)
Control level 1 (low)	0.91 ± 0.05	4.9	0.92 ± 0.04	4.6
Control level 2 (high)	18.53 ± 0.56	3.0	18.50 ± 0.53	2.7

Both the calculated CV values for repeatability and those provided by the manufacturer for both control samples are presented in Table 2.

**Table 2:** Repeatability of the measurement. x̄ - mean value; SD – standard deviation; CV – coefficient of variance.

Control level	Calculated statistical parameters			Statistical parameters provided by the manufacturer		
	x̄ (IU/L)	SD (IU/L)	CV (%)	x̄ (IU/L)	SD (IU/L)	CV (%)
Control level 1	0.87	0.027	3.15	0.69	0.03	4.10
Control level 2	18.36	0.637	3.50	18.44	1.09	5.05

The coefficient of variation for reproducibility (day-to-day variation) for both control samples was 2.2% and 2.6% for control levels 1 and 2, respectively. This meets the general minimum of analytical performance of laboratory methods (CV<5%) [29].

The within-laboratory measurement imprecision of the method and the total laboratory imprecision were calculated according to the recommended procedure [28] [30] [31] and presented in Table 3. Calculated CV for total intra-laboratory measurement imprecision for two commercial control samples for the IMMULITE®2000 TSI method lower than the ones provided by the manufacturer. Based on studies to date, there are no official data on biological variability for TSH-R-Ab, except for the reported imprecision and total measurement uncertainty data provided by the manufacturers of the two tests examined in this study [32].

**Table 3:** Within-laboratory imprecision.

Control level	Calculated statistical parameters			Statistical parameters provided by the manufacturer		
	Within-laboratory<br>measurement<br>uncertainty SD<br>(IU/L)	Total<br>measurement<br>imprecision CV<br>(%)	Extended<br>measurement<br>uncertainty (%)	Within-laboratory<br>measurement<br>uncertainty SD<br>(IU/L)	Total<br>measurement<br>imprecision CV<br>(%)	Extended<br>measurement<br>uncertainty (%)
Control level 1<br>(low)	0.028	3.23	6.46	0.030	5.00	10.00
Control level 2<br>(high)	0.699	3.81	7.61	1.265	6.35	12.70

The inaccuracy and systematic error of the IMMULITE®2000 TSI method were examined by evaluating the agreement between CLIA bridge immunoassay and ECLIA competitive binding immunoassay for determining TSH-R-Ab. A total of 86 samples whose TSH-R-Ab concentrations covered the clinically relevant measurement range were determined by both methods. The results showed a statistically significant correlation between the analysed methods (r=0.9041, p<0.0001). However, calculated statistical significance of p<0.001 indicates that the difference between the medians of the two groups of data is statistically significantly different from 0, i.e. that there is no statistically significant agreement between the results obtained by these methods. For 14 tested samples, a negative difference was obtained (the value obtained by the routine method was lower than that obtained by the IMMULITE®2000 TSI method). A positive difference was observed in 64 samples, and the same value was obtained for both tested methods in 3 samples.

**Figure 1:**
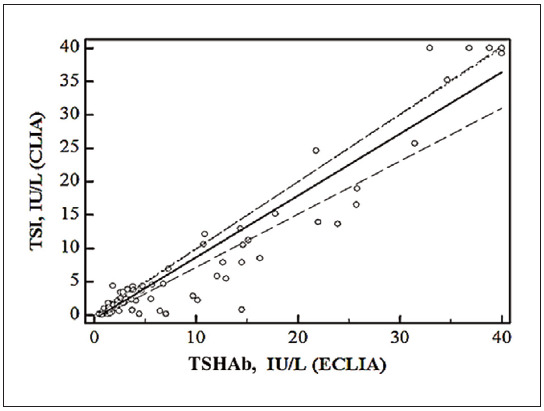
Passing-Bablok regression analysis comparing Elecsys Anti-TSHR Immunoassay (x axis) and IMMULITE®2000 TSI method (y axis).

Regarding method bias, the calculated bias was 2.32 IU/L, and the relative bias was 24.5%. Finally, the methods were compared using Passing and Bablok regression analysis (Figure 1). The following regression equation was obtained: y=0.63+1.11x, with a 95% confidence interval of 0.43 to 0.80 and 0.99 to 1.30 for the intercept and slope, respectively. The intercept value represents a constant, and the slope value reflects a proportional deviation in measurement. Since the obtained values of the 95%CI for the intercept do not include the value 0, we can conclude that there is a statistically significant difference between the value of the intercept and the value 0; that is, there is a constant deviation in measurement. The 95%CI for the slope includes the value 1, indicating no statistically significant deviation between the value of the slope and the value 1, indicating no proportional deviation in measurement between the examined methods.

After evaluating the analytical characteristics of the IMMULITE®2000 TSI bridge immunoassay, its diagnostic accuracy was examined, particularly in terms of sensitivity, specificity, and predictive values. To evaluate the diagnostic accuracy parameters of the IMMULITE®2000 TSI method, samples from control subjects were analysed in parallel to the patient samples. A positive measurement result, defined as a value above the cut-off value defined by the manufacturer (0.55 IU/L), was obtained in 72 out of 89 patients, which means that the sensitivity of the method tested, i.e. the ability of the assay to detect diseased populations, was 80.9%. A negative test result, defined as a value below the cut-off value set by the manufacturer (0.55 IU/L), was obtained in all 23 healthy volunteers. This result leads to the conclusion that the specificity of the tested method, defined as the ability of the test to detect healthy individuals correctly, was 100%. The PPV of the measurement (100%) was also calculated, which means there is a 100% probability that a person with a positive measurement result has the target condition (in this case, GO), i.e., that a positive result is really positive. The calculated negative predictive value was 57.5%, which suggests a 57.5% probability that a person with a negative measurement result is healthy, i.e., a 57.5% probability that a negative result is actually negative.

**Figure 2:**
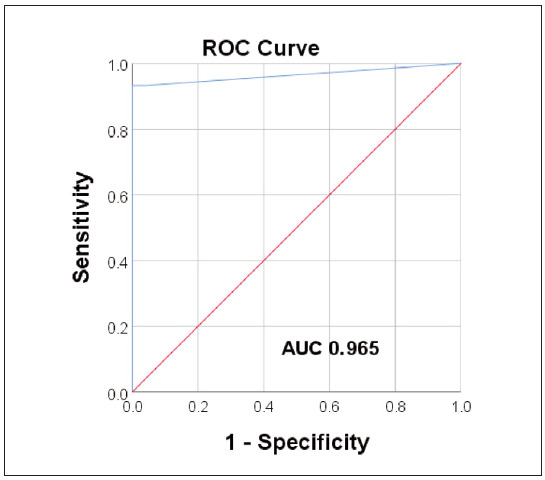
Receiver Operating Curve (ROC) analysis of the IMMULITE®2000 TSI immunoassay (AUC, area under the curve).

The diagnostic accuracy of the IMMULITE®2000 TSI method was further analysed using ROC analysis, that is, a combined analysis of the method’s sensitivity and specificity at different cut-off points. The ROC curve of the evaluated method is shown in Figure 2.

The value of the AUC ROC curve was 0.965 (p<0.001, for 95%CI: 0.932–0.997), which indicates an excellent diagnostic accuracy of the tested analytical method, that is, an excellent discriminatory ability to distinguish the diseased from the healthy population. The best ratio of sensitivity and specificity obtained by ROC analysis (93.3% and 100%, for sensitivity and specificity, respectively) was for a cut-off value of 0.1215 IU/L, which is a lower value compared to the cut-off value defined by the manufacturer (0.55 IU/L).

## Discussion

In this study, we comprehensively evaluated a relatively novel method (IMMULITE® 2000 TSI), currently not widely used in routine laboratory practice. Whenever an analytical method is implemented for the first time in a new laboratory environment, it is essential to verify its performance. This ensures that the analytical characteristics specified by the manufacturer can be reproduced under the local laboratory conditions. This process confirms whether the method meets the minimum criteria established during validation under the actual conditions in which it will be used [33].

The IMMULITE® 2000 TSI method uses the CLIA measurement principle in a bridge immunoassay format in which a chemiluminescent signal is detected proportional to the concentration of TSH-R-Ab present in the sample. According to the manufacturer, this test uses a recombinant human TSH-R (MC4), i.e., an epitope that is specific for TSAb [34]. However, the specificity of the bridging technology remains unverified, as several studies have reported non-specific TBAb detection by the MC4-expressing cell lines [22] [35] [36].

To date, two different groups of methods have been developed to measure TSH-R-Ab levels, namely immunoassays and bioassays [3] [7] [9]. Immunoassays reflect the antibody’s degree of binding to the receptor, which depends on several factors: the affinity and concentration of the antibody as well as the epitope structure [37]. Purified or recombinant TSH-R and monoclonal antibodies were used to determine TSH-R-Ab. Second-generation assays used bovine TSH, while the third-generation assays utilised a specific chimeric human-mouse monoclonal antibody. In the course of the further development of the immunoassay technology, only Siemens has presented a method for the non-competitive measurement of TSH-R-Ab. This method uses a pair of recombinant hTSH-R molecules. The TSH-R-Ab in the sample binds with one arm to the immobilised capture hTSH-R and with the other arm to the signal molecule, an alkaline phosphatase-labeled hTSH-R, creating a bridge-like structure [34].

On the other hand, bioassays can distinguish between stimulatory, neutral, and blocking autoantibodies based on their effects on cyclic adenosine monophosphate (cAMP) production in a stable cell line transfected with the receptor [8]. Although bioassays can distinguish TSH-R-Ab based on their function, practical aspects need to be considered. The introduction of this technology into routine laboratory practice is still very limited due to the time-consuming analytical procedure and the need for specialised laboratory equipment [16].

Almost two decades ago, third-generation immunoassays were introduced, showing significantly better clinical and analytical performance. Diagnostic sensitivity and specificity rose to 97.4% and 99.2%, respectively [30]. The determination of TSH-R-Ab, specific for GD, can significantly improve the early detection of the disease and allow for more specific monitoring and treatment. This emphasises the importance of evaluating the analytical properties of the method to ensure that the measurement result is accurate and precise. There is no officially published data on evaluating the analytical properties of the IMMULITE®2000 TSI immunoassay in laboratory practice in Serbia. However, previous studies indicate superior analytical performance compared to traditional competitive binding assays [37] [38] [39].

The results of this study indicate that a high degree of assay precision was achieved under routine laboratory conditions, as the CV values for repeatability and reproducibility were well below the values specified by the test manufacturer. The overall measurement uncertainty of the method was also significantly lower than that specified by the manufacturer. However, to date, there is no officially published data on the biological variability of TSH-R-Ab, so future studies will be required to investigate the bias and overall error of this method definitively.

Our intention in this study was to examine the concordance of a new IMMULITE®2000 TSI method with the currently widely used competitive binding immunoassay (Roche Elecsys Anti-TSHR Immunoassay). Our objective was to determine whether the new immunoassay format offers improvements in analytical and clinical performance. This routine method was chosen as the standard method routinely used in most clinical biochemistry laboratories. Another reason is that the results of both methods are expressed in the same units of the SI system (IU/L) and can be compared with each other to quantify the systematic measurement error under our laboratory conditions. Despite a statistically significant correlation of the results between the two methods studied, a significant deviation was found in the results obtained with these methods, with a relative bias of 24.5%. In addition, the regression analysis showed a constant deviation of the results obtained with the tested methods, which confirms that these two methods cannot be used for comparison, i.e. they cannot replace each other. The discrepancy obtained can be explained by the different detection principles of the tested methods and the different TSH-R epitopes used.

After evaluating the IMMULITE®2000 TSI method’s analytical properties, i.e., its clinical usefulness for the correct diagnosis and exclusion of GO, were investigated. For the clinical evaluation, samples from control subjects were analysed compared to samples from patients with GO. In this way, the ability of the tested method to detect both patients (diagnostic sensitivity of the method) and healthy subjects (diagnostic specificity of the method), i.e. its added value for clinical diagnosis, was determined. A positive result was obtained in 80.9% of the patients examined, indicating the method can diagnose 80.9% of people suffering from GO correctly. In other words, the IMMULITE®2000 TSI method showed a diagnostic sensitivity of 80.9% in the patient population studied. None of the control samples showed a positive result, indicating a diagnostic specificity of 100%, i.e. the ability of the test to exclude the diagnosis in 100% of healthy individuals. Compared to the high specificity of this method, an NPV of 100% was achieved, indicating that the diagnosis of GO can be excluded with a high degree of certainty in the case of a negative result. On the other hand, a relatively low PPV (57.5%) was determined, i.e. the probability that a positive result was obtained in a person with GO. A further ROC analysis showed that by lowering the cut-off value compared to the value stated by the manufacturer, the sensitivity-to-specificity ratio improved in the patient sample tested (93% and 100%, sensitivity and specificity, respectively), with an excellent AUC value of 0.965, indicating a high clinical value of the method tested.

In addition to addressing locally relevant scientific questions, this study’s strength lies in its settingwithin a reference university hospital in the tertiary healthcare sector. This allowed access to a large cohort of patients with severe GO forms under constant surveillance. The Clinic of Endocrinology, Diabetes, and Metabolic Diseases at the University Clinical Center of Serbia serves as a national reference centre for this type of pathology and accepts patients from all over the country. Considering the relatively low prevalence of GO in the general population, it can be assumed that a relevant number of patients were included in the study. The fact that the study was conducted in only one national centre is particularly important.

Investigating the affinity and dynamics of TSH-R-Ab using various biochemical assays could be valuable for improving diagnostic accuracy. Understanding the dynamics of these autoreactive antibodies offers important insights, particularly in cases where patients experience fluctuating hormonal and inflammatory states, which are frequently observed. It would be relevant to measure TSH-R-Ab activity at multiple time points to monitor their activity over a longer period and examine the correlation and concordance with the clinical outcome of the disease.

## Conclusion

Verifying the immunochemical IMMULITE 2000 TSI bridge immunoassay for TSH-R-Ab quantification confirmed an appropriate level of precision, which is essential for routine use. Additionally, a constant deviation between the two analysed methods indicated that these methods should not be used interchangeably. The fully automated IMMULITE TSI assay demonstrated a remarkable level of diagnostic performance potentially superior to the existing competitive-binding immunoassays, highlighting its potential for incorporation into efficient and cost-effective diagnostic workflows. However, further research is required to evaluate its analytical specificity.

## Dodatak

### Authors contribution

Neda Milinković and Marija Sarić Matutinović share the first authorship.

### Acknowledgements

This study has received funding from grant No. 175036 of the Ministry of Education, Science and Technological Development, Republic of Serbia, and through the Grant Agreement with The University of Belgrade–Faculty of Pharmacy No: 451-03-9/2021-14/200161.

### Conflict of interest statement

All the authors declare that they have no conflict of interest in this work.
